# Using social network analysis methods to identify networks of physicians responsible for the care of specific patient populations

**DOI:** 10.1186/s12913-022-07807-8

**Published:** 2022-04-08

**Authors:** Ronja Flemming, Wiebke Schüttig, Frank Ng, Verena Leve, Leonie Sundmacher

**Affiliations:** 1 Chair of Health Economics, Technical University of Munich, Georg-Brauchle-Ring 60/62, 80992 München, Germany; 2 Department for Health Services Management, Ludwig-Maximilian-University Munich, Munich, Germany; 3 Central Institute, for SHI Physician Care in Germany, Salzufer 8, 10587 Berlin, Germany; 4 Institute of General Practice (Ifam), Centre for Health and Society (Chs), Medical Faculty, Heinrich Heine University Düsseldorf, Moorenstr. 5, 40225 Düsseldorf, Germany

**Keywords:** Physician networks, Ambulatory care, Care coordination, Social network analysis, Quality of care

## Abstract

**Background:**

Coordinating health care within and among sectors is crucial to improving quality of care and avoiding undesirable negative health outcomes, such as avoidable hospitalizations. Quality circles are one approach to strengthening collaboration among health care providers and improving the continuity of care. However, identifying and including the right health professionals in such meetings is challenging, especially in settings with no predefined patient pathways. Based on the Accountable Care in Germany (ACD) project, our study presents a framework for and investigates the feasibility of applying social network analysis (SNA) to routine data in order to identify networks of ambulatory physicians who can be considered responsible for the care of specific patients.

**Methods:**

The ACD study objectives predefined the characteristics of the networks. SNA provides a methodology to identify physicians who have patients in common and ensure that they are involved in health care provision. An expert panel consisting of physicians, health services researchers, and data specialists examined the concept of network construction through informed decisions. The procedure was structured by five steps and was applied to routine data from three German states.

**Results:**

In total, 510 networks of ambulatory physicians met our predefined inclusion criteria. The networks had between 20 and 120 physicians, and 72% included at least ten different medical specialties. Overall, general practitioners accounted for the largest proportion of physicians in the networks (45%), followed by gynecologists (10%), orthopedists, and ophthalmologists (5%). The specialties were distributed similarly across the majority of networks. The number of patients this study allocated to the networks varied between 95 and 45,268 depending on the number and specialization of physicians per network.

**Conclusions:**

The networks were constructed according to the predefined characteristics following the ACD study objectives, e.g., size of and specialization composition in the networks. This study shows that it is feasible to apply SNA to routine data in order to identify groups of ambulatory physicians who are involved in the treatment of a specific patient population. Whether these doctors are also mainly responsible for care and if their active collaboration can improve the quality of care still needs to be examined.

## Background

Delivering high-quality health care is one of the chief aims of any health system. Doing so requires the organization and provision of care to be well coordinated and the relationship between providers and patients to be characterized by continuity. Especially among patients with chronic diseases or multimorbidity, ambulatory care that is continuous and well organized may reduce the incidence of negative outcomes, such as avoidable hospitalizations, or risks to patient safety, such as contraindicated treatments, and thereby enhance the quality of care [1–4]. The findings of Sundmacher et al. [5] suggest that, on average, 75% of hospitalizations resulting from certain ambulatory care-sensitive conditions, such as diabetes and heart failure, could have been prevented in Germany by better care coordination and continuity in the ambulatory care sector.

Achieving this, however, is one of the biggest challenges in the German health system. Almost all general practitioners (GPs) and about 45% of the specialists work in office-based practices [6], and patients are free to seek care from any of them without a referral [7]. There is no system of gatekeeping between primary and office-based specialist care, and patients can always obtain multiple opinions on a diagnosis and treatment [7]. Physicians do not necessarily learn about the treatments or diagnoses that their patients have received from other physicians unless the patient tells them or explicitly asks for their records to be transferred from one doctor to another. Efforts to improve this situation through digitalization have been hampered by the delayed introduction of an electronic health card for patients and a consequent lack of structured, digital documentation of the care they have received [7].

As in the rest of Europe, facilitated quality circles, also known as peer review groups, are a common method to manage and improve the quality of ambulatory care in Germany [8–12]. There is evidence that quality circles, for example, improve prescribing routines [13] or help GPs to conform more closely with good general practice criteria [14]. However, the participation of ambulatory care physicians in quality circles is voluntary and is not dependent on whether these physicians actually share a defined population or specific patients. Instead, these quality circles are organized more globally, either at a regional level or among specialties.

The Accountable Care in Germany (ACD) project aims to strengthen cooperation among ambulatory sector physicians in order to enhance the quality of care. The ACD project is funded by the German Federal Joint Committee (G-BA) from 2017 to 2021 as part of its innovation fund program. In contrast to the information gap faced by ambulatory physicians, routine data obtained by health insurance companies contain detailed information about the medical services provided to patients. Such data make it possible to follow the pathways that patients have taken through the health care system and identify the physicians who have provided them with treatment. One of the objectives of the ACD study was to apply social network analysis (SNA) to these data to identify interdisciplinary networks of ambulatory physicians responsible for the care of a shared population of patients.

SNA is used in many research domains to analyze social structures among connected individuals [15]. In the health care sector, this method is commonly applied to identify networks of health providers who do not necessarily share predefined geographic borders or specializations, but care for the same population of patients [16]. The methods for constructing these patient-sharing networks vary depending on the aims of the studies in which they are employed. Most studies of this nature have used routine data to identify all pairs of physicians who are connected through the patients they have in common, and to construct complex networks from these pairs [17–25]. Landon et al. [18] used SNA to identify networks of physicians who would be suitable candidates for building Accountable Care Organizations (ACOs) in the United States because of their shared patient group. They found that, when comparing community-based networks built with SNA with hospital-based networks regarding the proportion of care delivered within the networks, the former performed better. Thus, using SNA better reflected the actual physician visits than the hospital-based network identification and SNA is well suited to identify networks of physicians actually involved in the care of a shared patient population. Ostovari et al. [22] identified and analyzed the central providers in care teams of patients with diabetes mellitus and included physicians as well as pharmacists. They found that mainly medical laboratories and mail-order pharmacies were in central positions of the care teams, being connected to many other providers. In summary, SNA is applied to multiple research domains including health care settings using routine data. However, the method of SNA still lacks a consistent approach in the literature. We aim to provide a detailed framework for how to use SNA with routine data in order to identify networks with predefined properties including an exemplary application.

Applying SNA to routine data may help to organize quality circles by including physicians caring for the same patient population. Therefore, the ACD project applied this method to identify physicians with the same patient population to enhance quality of ambulatory care by strengthening collaboration.

Following a cluster randomized design, half the networks were subsequently invited to facilitated network meetings, akin to quality circles, in which we provided the participants with network information on the network characteristics and disease-specific indicators (such as hospitalizations) in order to facilitate discussions. The information was about both the treatment their shared patients had received and treatment pathways more generally [26].

The results of the intervention depend in large part on the construction of the networks, so this present paper aims to inform on the main challenges and decisions in the network construction process using SNA and its application in the studied project. With our work, we present and apply a framework for using SNA with routine data in order to derive groups of ambulatory physicians that have a selected patient population in common. We discuss the feasibility of applying the methodology to standard care and thereby highlight important decisions that need to be taken and point to potential pitfalls. The process described as an example in the ACD project can be transferred to other settings and systems by modifying the steps presented.

The remainder of the paper will therefore be structured as follows: after briefly describing the data requirements and the process of decision-making, we will present the stepwise methodology of network construction. The results comprise the presentation of the resulting networks and their characteristics. Finally, we will discuss the results in the context of the given study objectives.

## Methods

### Data

In Germany, approximately 90% of the population is covered by statutory health insurance (SHI) [27]. To identify networks, we used data from four regional associations of SHI physicians covering three of Germany’s 16 states: Schleswig–Holstein, Hamburg, and North Rhine-Westphalia. The dataset consisted of billing data from one year (2016–2017) for all physicians in these regions who were office-based and authorized to provide care to patients covered by SHI. All patients who consulted at least one of these physicians were included in our dataset. Patient and physician IDs were pseudonymized to guarantee privacy and ensure that all relevant data protection regulations were met. The dataset included information on patient diagnoses, age, and gender, as well as information on physician specialization, the types and billing codes of services provided, and the practice identification number.

### Decision-making process and study objectives

The decision-making process was conducted in an iterative manner, and conclusions were made by an expert panel that consisted of physician practitioners, routine data specialists, and health services researchers. Additionally, focus group interviews with physician practitioners were conducted, and a pilot study was carried out in order to test all elements of the study. The expert panel always made decisions based on the objective of the study, which targeted the implementation of facilitated network meetings in the ambulatory sector to improve quality of care. Based on this main objective, the research team identified the following four guiding study objectives, which were pursued in the network construction process:

A The final networks should be of a reasonable size (in terms of the total number of physicians) for implementing facilitated network meetings. It is assumed that not all network physicians would participate in the meetings.
B In order to ensure that network meetings can enhance the quality of care, the networks should include an interdisciplinary mix of ambulatory physicians who (a) are responsible for the care of a shared patient population and (b) actively participate in treating and coordinating the care of these patients.
C The networks should be responsible for the health care of a group of patients who (a) have conditions that are amenable, or sensitive, to ambulatory care and (b) require continuous and/or interdisciplinary treatment for optimal care and to avoid negative outcomes.
D For each network, a group of shared patients should be identified so that the physicians would be able to have informed discussions in the network meetings about the care that their common patients have received.

### Network construction

We identified five steps that generally need to be considered when constructing networks with SNA based on routine data. These steps include the following study-related aspects:

Step 1 Definition of units for network construction: Which health care providers (e.g., physicians, practices, or hospitals) should be connected with each other in a network, and what should be the basis for a connection (e.g., shared patients, referrals) between them?
Step 2 Definition of the health care provider population: Should specific health care providers (e.g., those from medical disciplines with only limited patient contact or from irrelevant regions) be excluded from network construction?
Step 3 Definition of the patient population: Does the study focus on a specific disease (e.g., diabetes, heart failure) and should certain patients (e.g., children, dialysis patients) be excluded from network construction?
Step 4 Network identification: Are there any predefined characteristics regarding network size (e.g., number of health care providers per network) or strength of connection (e.g., number of shared patients) the networks should achieve?
Step 5 Patient allocation to networks: Should patients be uniquely allocated to networks, and on which conditions (e.g., allocating patients based on the GP they are registered with or with some other predefined conditions)?

For each of these steps, important methodological considerations need to be taken into account, depending on the respective study objectives. In this section, we present and explain the informed decisions that were made for each step in general and as an exemplary application in the ACD project. These informed decisions, their rationale, and the technical details for each of the five steps are summarized in Table 1.

**Table 1:** Summary of the five steps of network construction and the informed decisions made by the expert panel in the ACD project

Step	Guiding study objectives		Informed decisions and explanations	Technical operationalization in the dataset
1. Units for network construction		**A**	We chose individual ambulatory physicians (and not practices) in order to identify all physicians involved in the patients' treatment Physicians from the same practice may also be interested in patients' pathways within their practice but also in other practices	Vertices are identified through the unique physician ID
2. Health care providers		**B**	Exclusion of physicians of predefined specializations and authorized physicians Reasons for exclusion: - Specialized in care of patients who are not included (e.g., children and adolescent specialists) - Specialists with no direct contact with patients do not play an active role but are mainly conducting contracted services (e.g., laboratory medicine or pathology) - Specialists providing mainly contracted services treat a large number of patients and would affect network construction (e.g., radiologists)	Excluded physician specializations: Children and adolescent specialists Laboratory medicine Microbiology Oral and maxillofacial surgery Pathology Radiology Radiation therapy Transfusion medicine
3. Patient population		**C**	Patients with one of 14 selected ambulatory care-sensitive conditions. Conditions are chronic or acute, have a high prevalence, and need continuous and/or interdisciplinary treatment We assigned patients to every physician who they consulted in a presumably "face-to-face" consultation and excluded selected types of billed services	Detailed information on operationalization is available in Table 1 Excluded types of billed services: Referral or billed service for a laboratory service as contract service; Request for laboratory service in a laboratory community Medical emergency service; Replacement during holiday or illness; Emergency; Emergency service with taxi; Rescue service; Central emergency service
4. Network identification		**B and D**	The minimum number of shared patients to define a connection between two physicians was set to 20 to ensure data protection. The 20 patients needed to account for at least 5% or more of the total patient population for at least one of the two physicians to ensure relevance The multilevel algorithm of the package igraph is used in an iterative manner to obtain networks of a size between 20 and 120 physicians	
5. Patient allocation		**C and D**	We defined a usual provider network for each patient who is mainly responsible for his or her care	We allocated patients to the network in which they had the most frequent physician consultations (in days). The number of consultations to one network had to exceed 50% of all physician consultations per patient

### Step 1 Definition of units for network construction

SNA is used in many fields of research and represents a powerful tool for analyzing complex and closely linked social structures. Networks usually consist of vertices connected by edges. The vertices may depict individuals, groups of people, or organizations [28]. The connections between them can originate from different sources, such as a friendship between two people or a commercial association between two organizations [15]. Therefore, we first defined the units for network construction, i.e., vertices and edges.

#### Exemplary application in the ACD project

In our analyses, the elements that are vertices are physicians. In line with previous research, a connection between physicians can be defined as the patients they have in common [16].

Owing to the large share of office-based physicians, organized primarily in solo or group practices, in the German ambulatory care sector, one approach could be to use practices as the network vertices. However, the expert panel decided to use individual physicians for multiple reasons. First, each physician has an individual role in the treatment of patients. Second, practices might be very large and include physicians with a variety of medical specialties, and not all of them participate in treating the same patients. Third, physicians from the same practice do not necessarily cooperate closely, and information about treatment might not automatically be transferred. Last, physicians from group practices might also be interested in information about the care received by their patients from other physicians. Therefore, considering physicians as vertices enables all physicians to participate in facilitated network meetings to strengthen cooperation and enhance the quality of care.

### Step 2 Definition of the health care provider population

After deciding upon the units for network construction, our next decision involved defining the population of health care providers, in our case ambulatory physicians who constitute the networks. This selection could include only a predefined group of physicians based on, for example, their regional location [25, 29] or their medical specialty [20].

#### Exemplary application in the ACD project

The overarching goal of the present study was to identify networks of an interdisciplinary mix of physicians (see study objective B) who are mainly responsible for the care of adult patients with ambulatory care-sensitive conditions. Specialists who are not frequently involved in the care of this patient population (e.g., children and adolescent specialists) or physicians who have only limited contact with patients (e.g., radiologists or pathologists) and therefore do not usually play an active part in coordinating continuous treatment will be excluded from the network construction. Additionally, the last mentioned group of specialists treats a large number of patients and might thereby bias network construction by establishing connections among physicians who may not have the same patients.

Another selection criterion was the regional location of the physicians. We always combined two regional associations of SHI physicians into larger intervention regions based on their geographic location, as patients are not required to see doctors in their own states. Therefore, of the four collaborating regional associations, Hamburg and Schleswig–Holstein (HH/SH) comprise one intervention region and North Rhine and Westphalia Lip (NO/WL) the other.

### Step 3 Definition of the patient population

The connections between two physicians were established through the patients they have in common. We specified the included patient population with regard to the study objective of identifying groups of physicians who are responsible for the care of adult patients with ambulatory care-sensitive conditions (see study objectives B and C).

#### Exemplary application in the ACD project

We used the provided list of diagnoses (International Classification of Diseases 10th revision codes, ICD-10) [30] from the German catalog for ambulatory care-sensitive conditions [5] and included all patients belonging to at least one of the first 14 diagnosis groups, which cause the highest number of hospitalizations (see Table 2). These 14 diagnosis groups include conditions that are ambulatory care-sensitive by definition, have a high prevalence in the population, and are mostly chronic. Additionally, these conditions require interdisciplinary and continuous treatment and are therefore relevant for study objectives B and C. In order to include all patients at risk of hospitalization, we extended the list of diagnoses by adding less severe conditions of these diagnoses. Dialysis patients were excluded because they are part of a large number of network connections due to frequent physician visits and might therefore bias the construction process.

In order to identify relevant physician visits in the selected patient population, we used available information in the billing dataset about the type of consultation. To ensure that only active and meaningful patient consultations are included, we focused on those that represent regular “face-to-face” visits and excluded, for example, consultations indicated as requests for laboratory service in a laboratory community.

**Table 2:** List of the 14 ambulatory care-sensitive diagnosis groups considered in the study

No	Diagnosis group	ICD-10	M2Q	M1Q		Diagnosis type
1a	Ischemic heart diseases	I20, I25	x			Status post AND/OR confirmed
1b	Ischemic heart diseases	I21, I22, I23, I24	x			Status post
2	Heart failure	I50	x			Status post AND/OR confirmed
3	Other diseases of the circulatory system	I05, I06, I07, I09, I08, I49, I48, I67, I70, I73, I78, I80, I83, I86, I87, I95, R00, I42, I74	x			Status post AND/OR confirmed
4a	Bronchitis	J20, J21, J22, J40, J41, J42, J43			x	Confirmed
4b	COPD	J44, J47	x			Status post AND/OR confirmed
5	Mental and behavioral disorders due to the use of alcohol or opioids	F10, F11	x			Status post AND/OR confirmed
6	Back pain [dorsopathies]	M42, M47, M53, M54, M50, M51	x			Status post AND/OR confirmed
7	Hypertension	I10–I15	x			Status post AND/OR confirmed
8	Gastroenteritis and other intestinal diseases	K52, K57, K58, K59			x	Confirmed
9	Intestinal infectious diseases	A00–A09			x	Confirmed
10	Influenza and pneumonia	J10, J11, J13, J14, J15, J16, J18, J12			x	Confirmed
11	Ear, nose, and throat infections	H66, J01–J03, J06, J31, J32, J35, H65, H73, J04, R07.0			x	Confirmed
12	Depressive disorders	F32, F33	x			Status post AND/OR confirmed
13	Diabetes mellitus	E10, E11, E13, E14, E16	x			Status post AND/OR confirmed
14	Gonarthrosis	M17	x			Status post AND/OR confirmed

*Notes*: Patients were assigned to one of the 14 diagnosis groups of more acute rather than chronic conditions (e.g., bronchitis) if they were diagnosed at least once with one of the ICD-10 codes during the observation period of 1 year (M1Q). These diagnoses needed to be labeled as “confirmed” in order to enhance coding accuracy. Conditions that are more chronic (e.g., hypertension) were identified if patients received at least two diagnoses in two different quarters during the observation period (M2Q). These diagnoses needed to be labeled as “confirmed” or “status post” after a hospital stay

### Step 4 Network identification

Comparing the patient population of every possible combination of two physicians, we identified the number of patients they have in common.^1^ This number was used to approximate the strength of the connection between two physicians, based on the assumption that physicians share more patients if they cooperate more closely [29].

Identifying all network connections between two physicians by comparing their patient populations may result in a dense and large network. Large networks can be split into smaller communities by applying modularity-based community detection algorithms [31]. These algorithms take into account the weighted connections between two physicians and optimize the modularity^2^ within and between networks by allocating physicians into smaller communities [31]. The network construction was conducted in R, and we used the multilevel algorithm [32] of the package igraph [33] because of its good performance with large datasets regarding computing time and accuracy [34].

#### Exemplary application in the ACD project

A minimum number of 20 shared patients was set as the first condition to define a connection between two physicians to ensure that the number of shared patients was large enough to be relevant for the informed discussions during the facilitated network meetings. The value of this threshold varies in the literature and depends on the objectives of the study and the size of the included patient population [16]. In this study, because, the underlying patient population of the 14 diagnosis groups accounted for more than 50% of the entire regional population, the threshold is higher than in studies analyzing only individual diseases [20, 22, 35, 36]. Additionally, because the project intended to report quality indicators about medical services provided by a network, the number of patients shared by two physicians needed to be sufficiently large to ensure data protection. A second condition presupposed that these 20 shared patients accounted for 5% or more of the total patient population for at least one of the two physicians. This relative threshold was intended to ensure that the number of shared patients represented a significant proportion for at least one of the two physicians’ patients.

The community detection was conducted in a multistage process to identify networks of appropriate size according to the objective of implementing facilitated network meetings (see study objective A). Taking into account that not all physicians will participate in the network meetings, the predefined network size was set to a minimum of 20 physicians with a maximum of 120. Applying the community detection algorithm can result in networks of any size, so the process was continued iteratively until networks of the predefined size were identified or until the networks could not be split further into smaller units. Smaller communities consisting of fewer than 20 physicians were excluded from further analyses.

### Step 5 Patient allocation to the networks

The network construction procedure results in a unique physician allocation, with each physician belonging to one network. In contrast, patients may be part of connections among a variety of networks. One has to decide upon patient belonging and can either let patients be part of many networks or allocate them to one specific network on account of specific predefined rules.

#### Exemplary application in the ACD project

The aim of the ACD study was to identify networks of physicians who are responsible for the treatment of a common patient population, and to enable informed discussions about their collaboration (see study objective D). The patients were thus each allocated to a network within which they had the largest number of physician visits (in treatment days). The network was considered to be the “usual provider network” only if this number of physician contacts with one network corresponded to more than 50% of all physician visits for a patient. All patients with the same usual provider network comprised the network’s patient population.

### Network statistics and correlation analysis

To understand the implications of the network construction parameters presented above, we calculated the correlation between selected network characteristics, which are important in the context of care delivery and network statistics, which describe the informal networks in a theoretical way. The network characteristics included the number of physicians and patients per network, the number of different medical specializations and practices comprised by each network, and the proportion of patient consultations within the networks. The network statistics included the degree centrality of the networks to capture how centralized networks’ care might be organized, the edge density as a measure of connectedness of physicians within the networks, and the clustering coefficient (transitivity) as a measure of network density [25]. The degree centrality was defined as the ratio of edges and vertices per network. The edge density was calculated as the number of existing edges divided by the total number of possible edges per network and can be interpreted as the degree of collaborative care realized in the network [24]. The transitivity measures the interconnectedness of the physicians within a network, which relates to the existence of local clusters. The transitivity considers the probability that two physicians with a common connected physician are connected with each other. It was calculated as the ratio of triangles and the existing triples in the network [33].

As the values of these metrics are often not normally distributed or ordinal, we calculated rank-based Spearman correlation coefficients. The calculated correlation values indicate the degree of the monotonic relationship between two variables, with values close to 1 indicating a strong positive association and values close to –1 indicating a strong negative association [37].

## Results

Overall, we identified 510 ACD networks consisting of the predefined number of physicians (20–120). These networks mostly present an interdisciplinary mix of physicians and thus fit the study objective to include a variety of relevant disciplines. The physician population in these networks was primarily influenced by the decision in Step 2 of network construction to focus on selected specializations. The following network construction steps resulted in some systematic exclusion of additional specialists, for example, because of the threshold of 20 shared patients to define a connection between two physicians.

The patients of the initial patient population, that was the basis for network construction, was in large part, multimorbid and had more than one of the 14 ambulatory care-sensitive diseases. Through the exclusion of certain patients in the steps of network identification and patient allocation, the final patient population had an even higher level of morbidity than the initial population.

In the following, we summarize the results of the network construction in the ACD project, focusing on the resulting networks, the included physicians, and allocated patients.

### Characteristics of the constructed networks

**Fig. 1:**
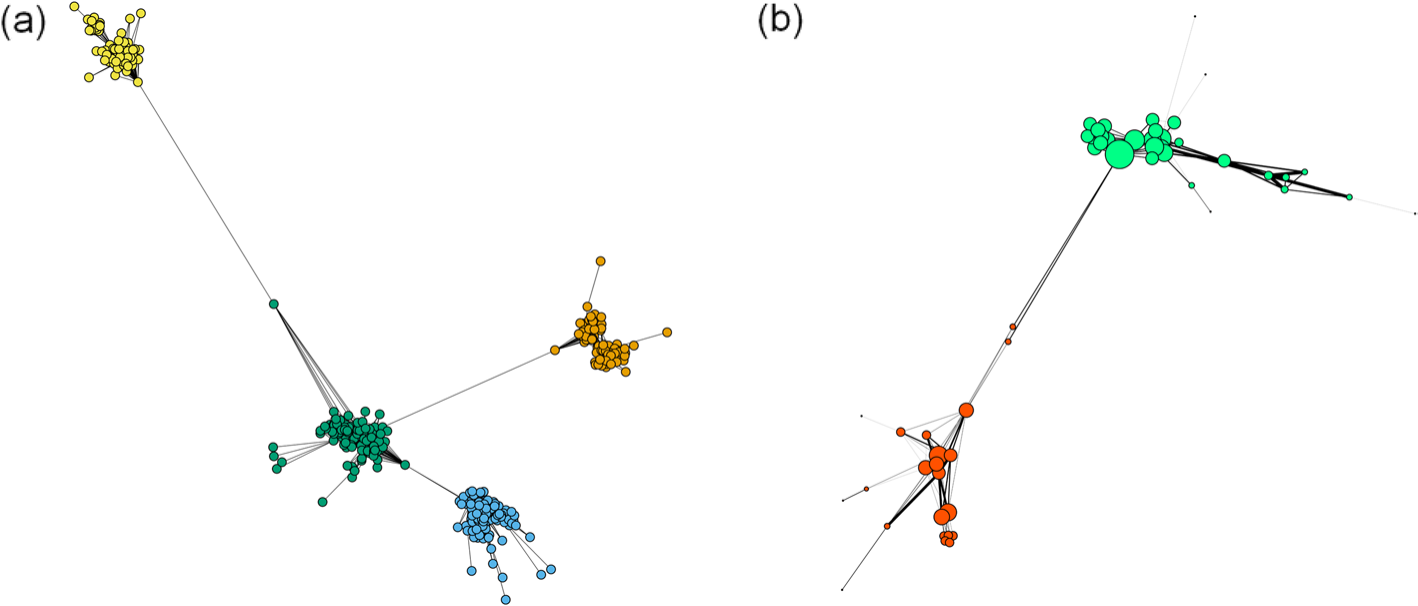
Exemplary figures of network structures. *Notes*: **a** The network comprises four communities identified by the multilevel algorithm. The vertices represent the physicians, the edges the shared patients, and the colors the four different communities identified by the algorithm. **b** This figure visualizes the strength of connection between two physicians: the thickness of edges is proportional to the number of shared patients. The size of vertices depicts the centrality of the physicians: the size is proportional to the degree of the physician (the number of connections to other physicians)

Figure 1 visualizes how the physicians (vertices) build the networks by being connected to each other and how the multilevel algorithm identifies communities of physicians who are connected more closely. The four identified communities in the exemplary figure (a) are visualized by different colored vertices. In Fig. 1b the thickness of edges additionally visualizes the strength of connection and the size of vertices the centrality (measured as the degree) of the physicians. The two communities depicted by different colors are only loosely interconnected and the two communities have more and stronger intra connections.

The network identification in Step 4 resulted in a total of 1,377 networks, of which 379 were from the intervention region HH/SH. Of these networks, 119 from HH/SH and 391 from NO/WL were of appropriate size for the ACD intervention. The rest were excluded because they comprised fewer than 20 physicians. The final 510 ACD networks included, on average, 52 physicians with a slightly smaller number of physicians per network in the intervention region in HH/SH, where the networks included 50 physicians on average. Even though most of the resulting networks comprised an interdisciplinary mix of physicians (i.e., 72% of networks included ten or more different specializations), there were different compositions of specializations included in the networks. For example, three networks included only physicians from two different specializations.

**Fig. 2:**
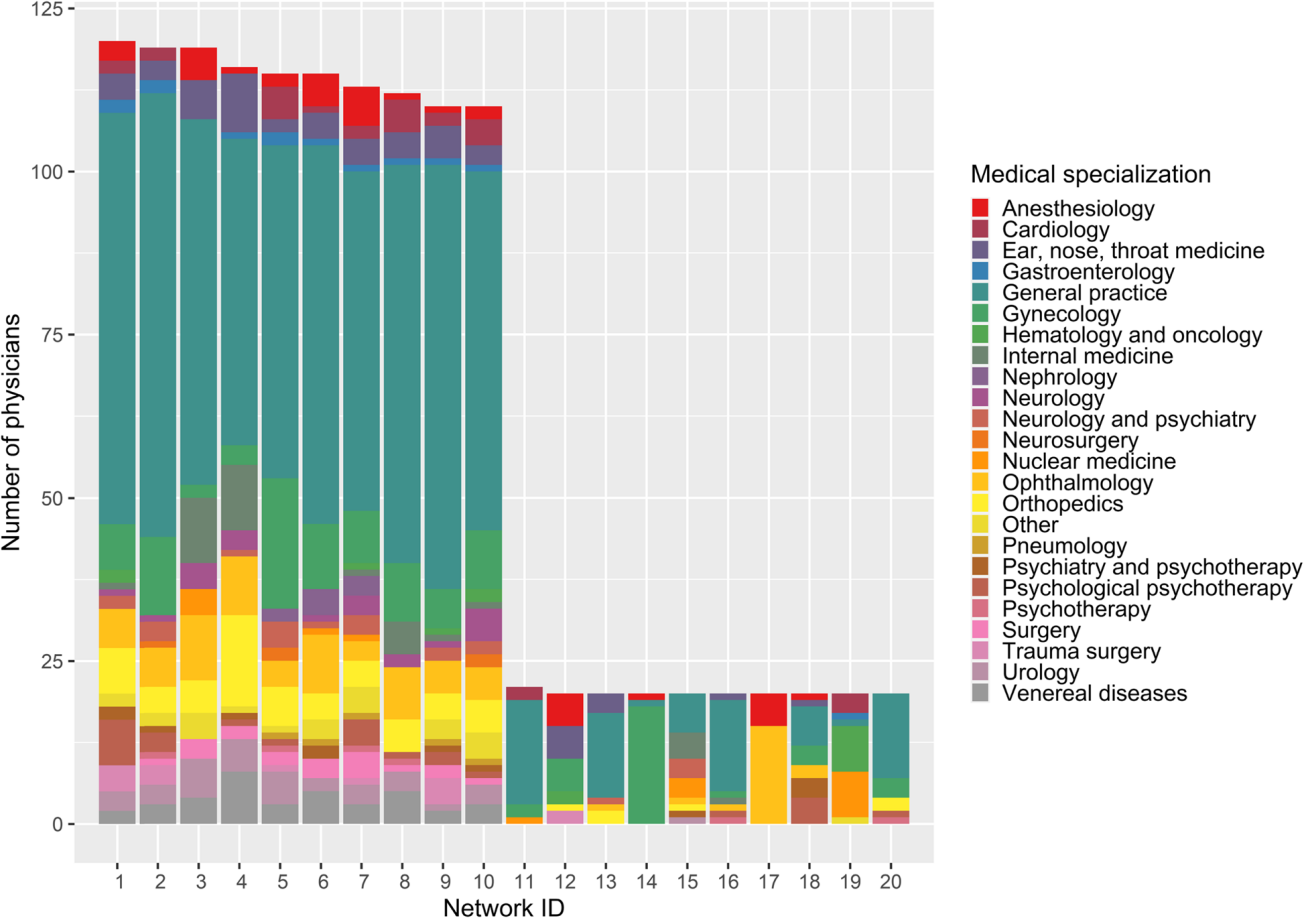
Specialization mix in the ten largest and smallest ACD networks.

An extract of the ten largest and smallest networks and their specialization composition is depicted in Fig. 2. In particular, the large networks consisted of physicians with numerous different specializations (more than 13), whereas the smaller networks included a maximum of eight. Further, in most of the depicted networks, the majority of physicians were GPs. This result held true for 89% of all the 510 ACD networks. However, there also existed some networks in which the majority of physicians were specialists. For example, the network ID 17 in Fig. 2 is comprised of 75% ophthalmologists and 25% anesthesiologists. Within the network construction, we based the network identification on individual physicians and not on practices. Consequently, some physicians from the same practices were allocated to the same networks and, in other cases, physicians from the same practice were allocated to different networks. On average, the ACD networks comprised physicians from about 35 different practices (31 in HH/SH and 36 in NO/WL), but there was one network from the intervention region HH/SH that consisted only of physicians from the same practice.

The connectivity among physicians within networks was explored by determining the number of shared patients between two physicians and the number of directly connected physicians per each network physician. On average, the network physicians who had an identified connection shared 165 patients (159 in HH/SH and 166 in NO/WL), and a network physician was connected to 14.8 other physicians from his or her own network (15.3 in HH/SH and 14.6 in NO/WL). The most central physicians in each network, defined by the largest number of connected physicians, were usually physicians for specialized care, e.g., ear, nose, and throat specialists, physicians for venereal diseases (both identified as central physicians in about 30% of the 510 networks), or ophthalmologists in 17% of networks.

### Physician population in the constructed networks

The definition of physician and patient population in the first three steps of network construction resulted in the initial physician database of 38,837 physicians. About 24% of physicians were from the intervention region HH/SH. Reducing this initial set of physicians to those who shared at least 20 patients with another physician resulted in a database of 32,000 physicians. In this step, particularly doctors with a rather small pool of patients treated per year, e.g., psychological psychotherapists were excluded.

**Fig. 3:**
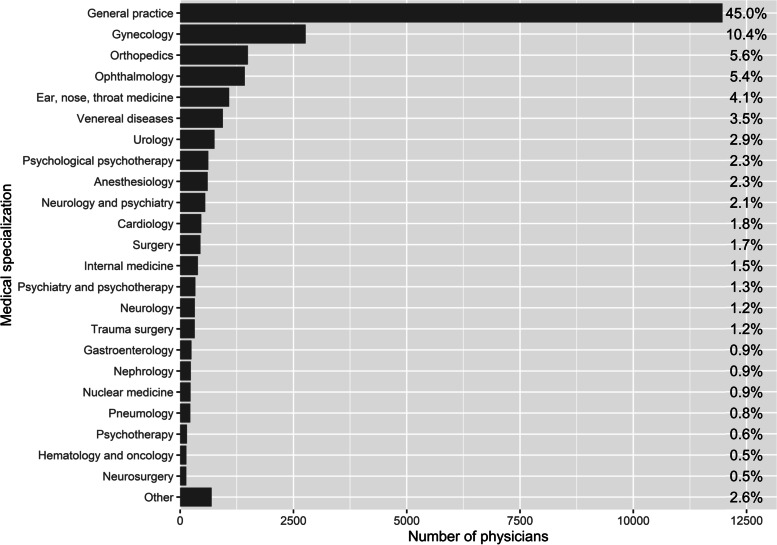
Relative and absolute numbers of physicians per specialization who are included in the 510 ACD networks

The subsequent network identification reduced the set of physicians to a final number of 26,581 physicians by excluding networks outside the defined size range. This exclusion did not systematically target any specific medical specialization. The distribution of medical specializations in the 510 ACD networks is depicted in Fig. 3. The most common physicians in the networks were GPs, who comprised 45% of the whole physician population. About 10% of physicians were gynecologists, while orthopedists and ophthalmologists each accounted for about 5%.

### Characteristics of the patient population included in the networks

In total, 12,113,444 patients with at least one ambulatory care-sensitive condition and complete age and gender information were treated by the network physicians, of whom 20% were residents in the intervention region HH/SH. The patient allocation to the 510 ACD networks in Step 5 reduced the initial patient population to a final number of 7,373,945 patients. The average age before and after allocation increased from 54.1 years to 55.8 years, and the proportion of women from 55 to 56% (see Table 3). The distribution of patients per diagnosis group is depicted in Table 3. The three largest diagnosis groups were hypertension, ear, nose, and throat infections, and back pain. The proportion of patients per individual disease group after allocation was slightly higher than in the initial patient population, as the average number of diseases per patient changed from 2.5 to 2.6. The proportion of patients with two or more diseases from the 14 selected disease groups increased from 67 to 71%. Thus, the final population had a higher level of morbidity than the initial patient population.

At the network level, an average of 14,459 patients was allocated to each network (12,060 in HH/SH and 15,191 in NO/WL). The smallest number of patients (95) was assigned to a network consisting of 20 physicians, and the largest number of patients in a network was 45,268 with the maximum possible number of 120 physicians. Table 3 shows that each network treated at least one patient from each of the 14 diagnosis groups. Thus, each network cared for a complete mix of patients.

**Fig. 4:**
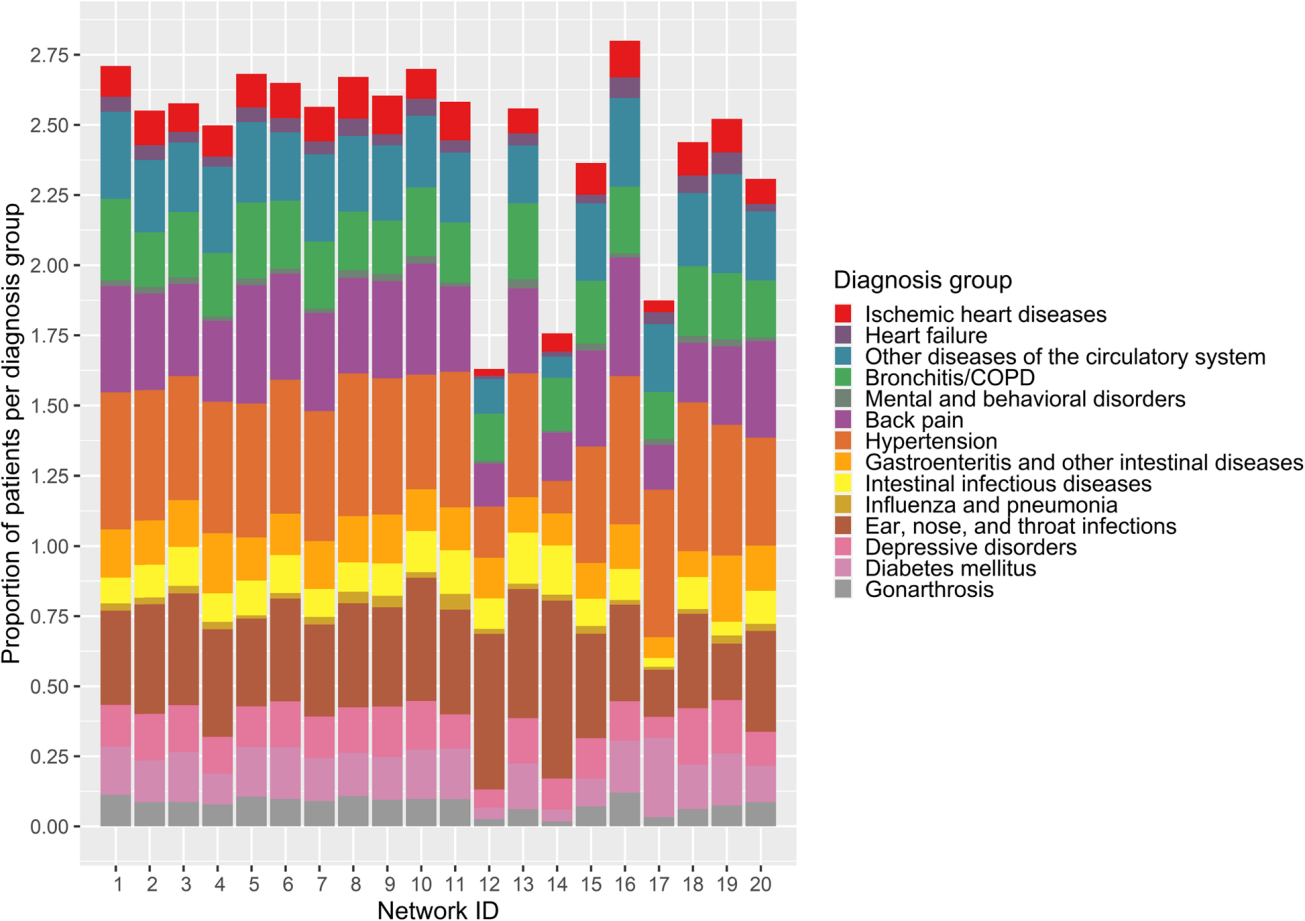
Relative numbers of patients per diagnosis group allocated to the ten largest and smallest ACD networks

Figure 4 displays the share of patients per diagnosis group for each of the ten largest and smallest networks. The relative distributions of diagnosis groups are similar for the depicted networks with some exceptions (networks with the IDs 12, 14 and 17): their allocated patients are less morbid on average than patients from the other networks.

Per the definition of patient allocation, in order to be assigned to a network, each patient needed to have more than 50% of all physician visits to physicians from that network. Averaged over patients, the proportion of visits to physicians within the network compared with all physician visits varied between 62.5% and 93.5% with an average of 79% over all networks. The percentage of physicians visited within the network varied between 33.2% and 87.2% per network.

**Table 3:** Summary statistics of the patient population

	Before allocation		After allocation in total		After allocation per network *n* = 7,373,945	
	Abs	Rel. (%)	Abs	Rel. (%)	Abs Mean [min; max]	Rel. (%) Mean [min; max]
Total	12,113,444		7,373,945		14,459 [95; 45,268]	
Ischemic heart diseases	1,206,318	10	847,928	11	1,663 [2; 5,525]	11 [0; 29]
Heart failure	510,614	4	360,797	5	707 [1; 2,404]	5 [0; 13]
Other diseases of the circulatory system	2,985,047	25	2,008,345	27	3,938 [23; 14,075]	26 [4; 67]
Bronchitis/COPD	2,942,435	24	1,839,672	25	3,607 [16; 13,145]	24 [9; 41]
Mental and behavioral disorders due to the use of alcohol or opioids	268,715	2	166,403	2	326 [2; 1,382]	2 [0; 48]
Back pain [dorsopathies]	4,240,119	35	2,745,598	37	5,384 [15; 17,642]	35 [8; 49]
Hypertension	5,052,074	42	3,379,043	46	6,626 [50; 22,041]	44 [3; 63]
Gastroenteritis and other intestinal diseases	1,840,768	15	1,167,217	16	2,289 [7; 7,776]	16 [7; 28]
Intestinal infectious diseases	1,455,880	12	867,106	12	1,700 [1; 5,656]	12 [0; 20]
Influenza and pneumonia	338,417	3	212,213	3	416 [1; 2,043]	3 [1; 12]
Ear, nose, and throat infections	4,749,687	39	2,792,413	38	5,475 [16; 16,305]	38 [17; 72]
Depressive disorders	1,960,498	16	1,165,462	16	2,285 [7; 8,403]	16 [4; 88]
Diabetes mellitus	1,778,080	15	1,218,905	17	2,390 [11; 7,801]	16 [1; 54]
Gonarthrosis	1,074,671	9	723,639	10	1,419 [3; 5,071]	9 [0; 16]
Age	54.06		55.78		55.4 [33.6; 71.5]	
Gender (female)	6,663,906	55	4,128,868	56	8,096 [51; 25,226]	56 [28; 100]
Number of diseases per patient	2.51 [1; 14]		2.64 [1;14]		2.58 [1.31; 3.15]

### Results of the correlation analysis of network characteristics and statistics

Table 4 summarizes the pairwise computed Spearman correlation coefficients between the reported network characteristics and statistics. The larger networks, measured in terms of the number of physicians, had a more diverse composition of medical specializations and a higher number of patients. Additionally, network size was positively associated with the frequency of patients consulting physicians within a network (Coef. (6) and (7) in Table 4).

The computed network statistics indicate that larger networks (related to the number of physicians or practices) were associated with a higher degree centrality and a smaller network density. The more central a network was organized (measured in degree centrality Coef. (8)) the higher the proportion of patients’ visits to physicians within the network compared to physicians from outside the network (Coef. (6) and (7)). Local clusters within the identified networks (Coef. (10)) occur more frequently in smaller networks and if the average number of physicians per practice in a network is high (Coef. (5)). Dense networks were associated with more local clusters.

**Table 4:** Results of the pairwise computed Spearman correlation coefficients

		(1)	(2)	(3)	(4)	(5)	(6)	(7)	(8)	(9)
		Spearman correlation coefficients (*p*-values)								
(1)	Number of physicians									
(2)	Number of patients	**0.837** (< 0.001)								
(3)	Number of different specializations	**0.822** (< 0.001)	**0.725** (< 0.001)							
(4)	Number of practices	**0.955** (< 0.001)	**0.850** (< 0.001)	**0.822** (< 0.001)						
(5)	Average number of physicians per practice	–0.028 (0.524)	–0.135 (0.002)	**–0.135** (< 0.001)	**–0.293** (< 0.001)					
(6)	Proportion of different physicians consulted within the network to all physicians	**0.593** (< 0.001)	**0.788** (< 0.001)	**0.629** (< 0.001)	**0.563** (< 0.001)	0.051 (0.252)				
(7)	Proportion of physician consultations within the network to all physician consultations	**0.552** (< 0.001)	**0.811** (< 0.001)	**0.598** (< 0.001)	**0.557** (< 0.001)	–0.057 (0.196)	**0.953** (< 0.001)			
(8)	Network degree centrality	**0.559** (< 0.001)	**0.727** (< 0.001)	**0.631** (< 0.001)	**0.533** (< 0.001)	0.020 (0.656)	**0.914** (< 0.001)	**0.869** (< 0.001)		
(9)	Network density	**-0.379** (< 0.001)	-0.064 (0.148)	**-0.148** (< 0.001)	**-0.357** (< 0.001)	0.011 (0.807)	**0.367** (< 0.001)	**0.358** (< 0.001)	**0.500** (< 0.001)	
(10)	Clustering coefficient	**-0.299** (< 0.001)	-0.074 (0.096)	**-0.147** (< 0.001)	**-0.335** (< 0.001)	**0.157** (< 0.001)	**0.344** (< 0.001)	**0.296** (< 0.001)	**0.437** (< 0.001)	**0.827** (< 0.001)

*Notes*: The values in bold indicate a significant correlation between the characteristics at a 0.1% significance level (*p* < 0.001)

## Discussion

The aim of this study was to provide a framework for and investigate the feasibility of applying SNA to identify networks of ambulatory care physicians. This was done within the context of the ACD project, which aimed to identify groups of ambulatory health care providers who are jointly responsible for the treatment of patients with ambulatory care-sensitive conditions. We presented a network construction framework consisting of five steps that can be used to apply these SNA methods and demonstrated its application within the ACD project. During the second phase of the ACD project, the identified network physicians were then invited to facilitated network meetings to enhance quality of care by strengthening collaboration.

The results of this study confirm the feasibility of using the presented framework in order to identify groups of ambulatory physicians in standard care who have patients in common. The ACD networks were constructed based on the predefined requirements, which were established to address the study objectives. However, each decision within the network construction steps was accompanied by specific consequences that need to be taken into account when planning to use SNA with routine data. These findings are summarized briefly as follows:

### Step 1 Definition of units for network construction

The first decision about the definition of units for network construction was to use individual physicians and not their practices. The results indicate that this definition led to a certain variability in the composition of networks. Hence, some networks consisted of physicians from different practices, and others had a large proportion of physicians representing the same practice. For the ACD project, this result does not conflict with the study objectives. However, constructing networks based on practices maybe useful in some cases, for example, when physicians from the same practice should generally be included in the same network or when collaboration among practices should be analyzed.

### Step 2 Physician population

The exclusion of physicians with predefined specializations allowed us to build a homogeneous group of physicians. One reason for excluding specialists, such as radiologists, was to prevent them from biasing the network constructing process because they are connected to many physicians and do not necessarily play a participative role in patients’ care coordination. The results indicate that specialist physicians (e.g., ear, nose, and throat specialists, physicians for venereal diseases, and ophthalmologists) were at the center of many networks, with the center being defined as having the highest number of connections with other physicians. Thus, excluding the specialists who have only little patient contact and conduct mainly contract services was a reasonable decision.

Having specialist physicians at the center of networks might not represent the assumed structure of health care networks from a patient’s perspective, which might assume that GPs would be in this position, coordinating the patients’ care. However, for the ACD project, this was not a major concern, because all network members were invited to facilitated network meetings, and their location within their network was not of primary interest.

### Step 3 Patient population

The predefinition of certain patient population characteristics ensured that the identified groups of health care providers are responsible for and connected through patients with ambulatory care-sensitive conditions. Effective ambulatory treatment and good management of chronic conditions can reduce the risk of hospitalizations for these patients. Therefore, active cooperation among network physicians treating this patient population is particularly important. The results further indicate that patients allocated to the networks had an average of more than one of the 14 diseases, demonstrating that collaboration among an interdisciplinary mix of physicians is crucial for this population.

### Step 4 Network identification

The results demonstrate that the number of different specializations per network was, on average, sufficiently large and that, in turn, each network treated a sufficiently large number of patients in common. However, the medical specialties represented within the networks depended on the definition of the minimum number of shared patients. Physicians with only a small patient population were systematically excluded for data protection reasons. Nevertheless, it would have been more appropriate to set the threshold to a smaller number or to use only a relative threshold in order to include the complete set of physicians consulted by the patient population.

The iterative manner of applying the network detection algorithm resulted in a sufficient number of networks of the appropriate size for the intervention study. This procedure did not lead to systematic exclusions of physician specializations. Regional differences in physician density and their interconnectivity in patient-sharing networks might have influenced the identification of networks and their size.

### Step 5 Patient allocation to networks

The patient allocation in Step 5 reduced the set of patients by 40% but ensured the active participation of network physicians caring for this pool of patients. Dependent on the study objectives, a unique allocation of patients to networks might not be necessary but, for the ACD project, the identification of health care providers who are mainly responsible for the patients’ treatment was a major objective. The variability in proportions of visits to physicians within and outside the networks observed in our results suggests that such a normative specification (50% of physician consultations within the network) for patient allocation is necessary in order to ensure responsibility for care at the network level.

On average, the patient allocation also resulted in networks of patients with higher morbidity levels compared with the initial patient population. This result might lead to a focus on patients with a particular need for continuous treatment, where cooperation among their physicians would have the potential to avoid negative health outcomes.

Summarizing the results of this research project, one needs to consider some limitations. First, in general, routine data are collected for purposes of reimbursement. They therefore have some properties that might have led to inaccuracies in the present study: the identification of real “face-to-face” contacts between patients and physicians could only be approximated by focusing on selected types of billing positions. Whether this selection in real life depicts those consultations we wanted to include is not clear. Additionally, variations in billing habits and the coding of diagnoses in the ambulatory care sector complicated a precise definition of patient–physician consultations and of the patient population through their diagnoses. Second, we do not provide exhaustive sensitivity analysis but focus on predefined objectives and their degree of achievement. This might not have led to one analytically best solution but ensured the feasibility of the intervention study.

Last, our results show that, even though all the objectives could be met, there also existed networks with extreme values for their characteristics, as for example, three networks comprising only physicians from two different medical specialties. Thus, variation among the characteristics needs to be tolerated, or further specifications need to be considered.

## Conclusions

Identifying informal networks of ambulatory physicians who care for a common population is especially challenging in systems in which patients can choose their physicians freely. At the same time, collaboration within such networks may lead to improvements in the quality of care. The present study shows that SNA makes it possible to identify informal networks of ambulatory physicians caring for the same patients using routine data. However, this method still lacks a consistent approach in the literature. We provide a detailed framework for how to use SNA with routine data in order to identify networks with predefined properties. Our results demonstrate that the predefined objectives in the ACD study could be met. We additionally provide evidence and insights into how theoretical social network statistics correlate with characteristics describing the informal networks in ambulatory care, such as the composition or size of networks. With some adaptations, the procedure may be applied to other settings and data structures. Future research could focus on the methodological part of this study and could systematically investigate how changes in the steps for network construction impact upon the metrics analyzed within this study. One could further focus on single disease groups and analyze whether and how the informal networks differ depending on the patient group building the informal networks.

In order to make use of the provided findings in ambulatory care it would be necessary evaluating the identified networks concerning their responsibility of care and the effect of implementing facilitated network meetings.

## Data Availability

The data that support the findings of this study are available from the Regional Association of SHI Physicians Hamburg, the Regional Association of SHI Physicians Schleswig–Holstein, the Regional Association of SHI Physicians Westphalia Lip, and the Regional Association of SHI Physicians North Rhine but restrictions apply to the availability of these data, which were used under license for the current study, and so are not publicly available. Data are however available from the authors upon reasonable request and with permission of the Regional Associations and their supervisory authorities.

## Abbreviations

ACD: Accountable Care in Deutschland
ACO: Accountable Care Organization
GP: General practitioner
HH/SH: Intervention region Hamburg/Schleswig–Holstein
NO/WL: Intervention region North Rhine/Westphalia Lip
SNA: Social network analysis
SHI: Statutory health insurance

## Footnotes

1 A patient was counted as being treated by both physicians if he or she had at least one visit to each physician during one year (see explanation in Table 1, Step 3).

2 The modularity of a community is high if the occurrence of edges within the community is higher than would have been expected in the case of a random distribution of the edges.
